# Pre-Clinical Studies with D-Penicillamine as a Novel Pharmacological Strategy to Treat Alcoholism: Updated Evidences

**DOI:** 10.3389/fnbeh.2017.00037

**Published:** 2017-03-07

**Authors:** Alejandro Orrico, Lucía Martí-Prats, María J. Cano-Cebrián, Luis Granero, Ana Polache, Teodoro Zornoza

**Affiliations:** ^1^Área de Investigación en Vacunas, Fundación para el Fomento de la Investigación Sanitaria y Biomédica de la Comunidad Valenciana (FISABIO)Valencia, Spain; ^2^Department of Psychology, University of CambridgeCambridge, UK; ^3^Department of Pharmacy and Pharmacy Technology and Parasitology, University of ValenciaValencia, Spain

**Keywords:** D-penicillamine, pre-clinical studies, acetaldehyde sequestering agent, ethanol relapse prevention, voluntary alcohol consumption

## Abstract

Ethanol, as other drugs of abuse, is able to activate the ventral tegmental area dopamine (VTA-DA) neurons leading to positively motivational alcohol-seeking behavior and use, and, ultimately to ethanol addiction. In the last decades, the involvement of brain-derived acetaldehyde (ACD) in the ethanol actions in the mesolimbic pathway has been widely demonstrated. Consistent published results have provided a mechanistic support to the use of ACD inactivating agents to block the motivational and reinforcing properties of ethanol. Hence, in the last years, several pre-clinical studies have been performed in order to analyze the effects of the sequestering ACD agents in the prevention of ethanol relapse-like drinking behavior as well as in chronic alcohol consumption. In this sense, one of the most explored interventions has been the administration of D-Penicillamine (DP). These pre-clinical studies, that we critically summarize in this article, are considered a critical step for the potential development of a novel pharmacotherapeutic strategy for alcohol addiction treatment that could improve the outcomes of current ones. Thus, on one hand, several experimental findings provide the rationale for using DP as a novel therapeutic intervention alone and/or in combination to prevent relapse into alcohol seeking and consumption. On the other hand, its effectiveness in reducing voluntary ethanol consumption in long-term experienced animals still remains unclear. Finally, this drug offers the additional advantage that has already been approved for use in humans, hence it could be easily implemented as a new therapeutic intervention for relapse prevention in alcoholism.

## Introduction

In the last years, numerous studies have supported the idea that, at least in part, motivational and neuropharmacological effects of ethanol are mediated by its first brain-derived metabolite, acetaldehyde (ACD) and/or its bioderivates (for extensive review, see Deehan et al., 2013; Peana et al., 2016). The most widely employed strategy to demonstrate the involvement of ACD in the motivational and reinforcing effects of ethanol has been, for years, the pharmacological manipulation of the enzyme system activity implicated in the brain metabolism of this drug. For instance, modulating catalase (Correa et al., 1999, 2004; Sanchis-Segura et al., 1999a,b; Arizzi-LaFrance et al., 2006; Font et al., 2008), cytocrome P-4502E1 (Hipolito et al., 2009; Ledesma et al., 2014) or alcohol dehydrogenase (Escarabajal and Aragon, 2002) brain activity.

Furthermore, another strategy has allowed disentangling the role of ACD in different behavioral effects induced by ethanol: the use of ACD sequestering agents. Fortunately, ACD is a highly reactive molecule and, therefore, capable of being “sequestered” by thiol amino acids such as L-cysteine (L-cys) and D-penicillamine (DP), which react non-enzymatically with ACD to form stable non-toxic adducts. This fact has been evidenced not only *in vitro* (Nagasawa et al., 1980; Kera et al., 1985) but also *in vivo* (Serrano et al., 2007) experimental conditions. Interestingly, these compounds, besides being used in the aforementioned research strategy, could have other advantages, particularly from a clinical point of view. In this sense, these ACD-scavenging compounds would not alter neurotransmitter systems, thus avoiding the manifestation of unexpected side effects displayed by the most promising candidates which have been evaluated in pre-clinical studies (Salaspuro et al., 2006; Leggio et al., 2010). They act removing/blocking both hepatic and brain-derived ACD, thus potentially preventing the reinforcing and motivational properties of ethanol-derived ACD on specific regions and pathways of the brain. Behavioral studies have demonstrated that DP is able to: (i) dose-dependently prevent the ethanol- and ACD-induced conditioned place preference (CPP) in rodents (Font et al., 2006a,b; Peana et al., 2008, 2009); (ii) attenuate either behavioral depression caused by ACD or behavioral locomotion induced by ethanol in mice (Font et al., 2005); and (iii) prevent, in a dose-dependent manner, the motor activation induced by intra-ventral tegmental area (VTA) ethanol administration (Martí-Prats et al., 2010, 2013). Additionally, neurochemical studies have evidenced that this drug suppresses both ethanol- and ACD-induced stimulation of dopamine (DA) release in the nucleus accumbens shell as well as the ethanol-evoked excitation of VTA-DA neuron activity (Enrico et al., 2009). Moreover, in most of these articles, the specificity of DP effects has been addressed using other drugs of abuse such as cocaine, caffeine or morphine.

Considering these promising results, several groups have explored the pre-clinical validity of ACD inactivation with DP as an alternative strategy for the development of new pharmacological approaches for treatment of alcoholism. Hence, the effect of DP in the prevention of ethanol relapse-like drinking behavior as well as in voluntary alcohol consumption have been repeatedly demonstrated (Font et al., 2006b; Orrico et al., 2013; Martí-Prats et al., 2015). In addition, the fact that DP is currently approved by the Food and Drug Administration (FDA) and European Medicines Agency (EMA) for other indications, offers the additional advantage to its immediate potential clinical utility.

In this review article, readers will find a compilation of the most remarkable publications in which the potential use of DP in the prevention of ethanol relapse-like drinking behavior as well as in voluntary alcohol consumption has been investigated in a pre-clinical environment.

### Effect of DP in Relapse-Like Drinking Behavior

Among the few animal models of relapse presently available, the Alcohol Deprivation Effect (ADE—a marked increase in ethanol consumption that follows periods of deprivation) has become a widely used model to examine the efficacy of potential medication providing excellent face and predictive validity (Rodd et al., 2004; Spanagel and Kiefer, 2008; Spanagel, 2009; Bell et al., 2012). For instance, the three medications currently used in the clinical setting—Acamprosate, Naltrexone (NTX) and Nalmefene—, although they have different pharmacological mechanisms of action, have all been proven to effectively reduce the ADE in rodents (Spanagel and Zieglgänsberger, 1997; Orrico et al., 2014; Spanagel et al., 2014; Vengeliene et al., 2014). Nowadays, Acamprosate’s primary mechanism of action still remains unclear, although it is believed to normalize the balance between excitatory and inhibitory pathways throughout the glutamatercic system (De Witte et al., 2005). On the other hand, NTX as well as Nalmefene work as opioid antagonists at μ and δ receptors and as agonists at κ receptors (Wackernah et al., 2014).

The ADE phenomenon can be triggered under both operant (Hölter et al., 1997; Echeverry-Alzate et al., 2012) and home-cage free choice drinking, non-operant, conditions (Spanagel and Hölter, 1999; Vengeliene et al., 2014). In this sense, along the last years, our group has extensively employed this latter model to explore, for the first time, the potential role of DP in ethanol relapse prevention.

#### Non-Operant Procedures

**Figure 1:**
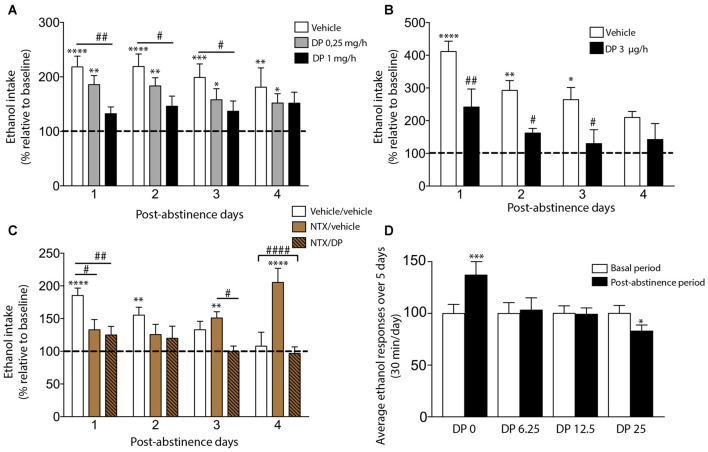
**Effect of D-penicillamine (DP) on alcohol deprivation effect (ADE) measurements under both non-operant (A–C)** and operant paradigms **(D)**. **(A)** Subcutaneous DP treatment dose-dependently suppresses the ADE manifestation. Effect of subcutaneous infusion of vehicle or DP (0.25 or 1 mg/h) on ethanol intake. **(B)** Intra-ventral tegmental area (VTA) infusion of DP blocked the ADE expression. Effect of the intra-VTA infusion of vehicle or DP 3.0 μg/h on ethanol intake. **(C)** DP prevents the “delayed ADE” induced by the continuous blockade of the opioid receptor with Naltrexone (NTX). Effect of vehicle, NTX or NTX/DP administration on ethanol intake. In panels **(A–C)**, animals had 9–11 months of ethanol experience. The percentage of each rat’s total daily ethanol intake during post-abstinence drinking days was calculated with respect to baseline drinking just before deprivation (dashed line). The basal alcohol intake was about 0.6–1 g/kg/day. The deprivation period lasted 14 days. The assayed treatments began 48 h before the reintroduction of ethanol bottles (available 24 h along the post-abstinence period). **(D)** DP is able to prevent the ADE under an operant paradigm. Average ethanol responses in animals intraperitoneally (i.p.) administrated with saline (DP 0) or DP (6.25, 12.5 and 25 mg/kg), 30 min before each self-administration session in the post-abstinence period. Asterisk denotes statistically significant differences relative to the respective baseline period (*p < 0.05; **p < 0.01; ***p < 0.001; ****p < 0.0001). *Post hoc* test showed differences between groups (^#^*p* < 0.05, ^##^*p* < 0.01, ^####^*p* < 0.0001; Adapted from Orrico et al., 2013, 2014; Martí-Prats et al., 2015).

Our recent work has successfully shown that DP is able to prevent the ADE in Wistar rats using a home-cage four-bottle free choice (water, ethanol 5%, 10% and 20%) paradigm (Orrico et al., 2013, 2014). Concretely, at the end of the fifth deprivation period and 48 h before the reintroduction of ethanol bottles, rats were subcutaneously (SC) implanted with a mini-osmotic pump delivering at a constant rate either vehicle or DP (1 or 0.25 mg/h), during 1 week. The results obtained demonstrated that DP dose-dependently prevented the ADE in long-term ethanol-experienced rats. In fact, the constant-rate SC infusion of DP at a dose of 1 mg/h, but not 0.25 mg/h, completely prevented the ADE phenomenon (Figure 1A), while the vehicle-treated group increased the ethanol intake along the four post-abstinence days compared to baseline. Hence, our data clearly indicate that systemic administration of DP is able to prevent the expression of the ADE without affecting total fluid consumption and body weight (Orrico et al., 2013).

In spite of these results, one important concern should be considered. As can be seen in detail in the following section, some studies have demonstrated that systemic, but not intracerebroventricular (ICV), administration of DP is able to produce changes in the taste of fluid and food (Font et al., 2006a). Hence, the possibility that, at least, part of the preventive effects of DP on the ADE could be produced by an alteration in taste perception cannot be fully ruled out. Nonetheless, and in agreement with these results, our group also demonstrated that intra-posterior VTA (pVTA) administration of DP was able to suppress the ADE (Figure 1B), suggesting that the preventive effects of DP on ADE could, at least in part, be caused specifically by a mechanism independent of taste alteration (Orrico et al., 2013).

Most drugs of abuse, including ethanol, stimulate the release of DA in several limbic regions (Di Chiara, 2002). In recent years, it has been shown that ACD is a crucial component of the overall effects of ethanol on DA neurons of the VTA (Rodd-Henricks et al., 2002; Rodd et al., 2005, 2008; Melis et al., 2007; Deehan et al., 2013). Therefore, our results also showed, for the first time, that pVTA is a critical region for DP action in relapse-like drinking behavior and emphasize the role displayed by this brain area in the relapse phenomenon.

The abovementioned results encouraged us to further study the potential use of DP alone or in combination with other marketed drugs (such as NTX) as a promising strategy to increase the efficacy of current anti-relapse therapies, based on the neurochemical studies that have confirmed that the mechanism through which ethanol, or more probably ACD, excite DA neurons is dependent on the Mu-Opioid Receptors (MORs; Sánchez-Catalán et al., 2009; Peana et al., 2011). Accordingly, our group next conjectured that the NTX/DP combination, due to its distinct but complementary mechanisms of action to impede MORs activation could be more efficacious in ADE prevention (Orrico et al., 2014). Specifically, we explored whether the combination of DP with NTX could suppress the delayed ADE, i.e., the rebound in alcohol consumption detected in animal laboratory models after continuous blockade of the opioid receptor with antagonists such as NTX (Heyser et al., 2003) or naloxone (Hölter and Spanagel, 1999). In this sense, in several strains of rats using free choice paradigms it has been demonstrated that NTX decreases ethanol consumption (Overstreet et al., 2006), however, tolerance to this effect was demonstrated after repeated drug administration, leading to an increase in alcohol consumption (Cowen et al., 1999; Juárez and Eliana, 2007). This fact is probably due to the MORs up-regulation associated with its continuous blockade (Hyytiä et al., 1999; Overstreet et al., 1999; Orrico et al., 2014). Thus, as can be seen in Figure 1C, in our experimental conditions, NTX powerfully blocked the ADE on post-abstinence days 1 and 2 in agreement with previously published data, but an increase in alcohol consumption, with respect to basal values, was detected on post-abstinence days 3 and 4 (the manifestation of the so-called “delayed ADE”). The results obtained supported the efficacy of the NTX/DP combination preventing not only the ADE expression, but also the delayed ADE. In fact, the combination of DP (0.25 mg/h; a non-effective dose in our previous article) and NTX (2 × 5 mg/kg SC per day) showed an adequate anti-relapse pre-clinical efficacy along the four post-abstinence days.

In summary, the reported data demonstrate that this therapeutic strategy, of combining two drugs with complementary actions—opioid receptor blockade (by NTX) and chemical ACD inactivation (by DP), shows adequate alcohol anti-relapse-like drinking efficacy in long-term ethanol-experienced rats. Moreover, it overcomes some therapeutic limitations of either drug alone, since this combination is able to block not only the delayed increase in ethanol consumption, typically occurring after chronic opioid antagonist administration, but it also allows the administration of sub-threshold DP doses. All in all, these findings suggest that sequestering agents of ACD, in general, and DP, in particular, may represent a valuable therapy in the management of relapse in alcohol-dependent patients.

#### Operant-Procedures

There is no doubt among researchers that in order to maximize the translational power of pre-clinical research, it is important to gather evidence for as many paradigms and different animal models as possible (Bell et al., 2012). Hence, the use of different paradigms to test the same observations would assure the reproducibility of pre-clinical data, which is a challenge for neuroscience (Steckler, 2015).

In this context, we were able to validate our previous work on the pre-clinical efficacy of DP using a different laboratory paradigm: an operant procedure. Several authors have used this operant paradigm to demonstrate the capacity of a number of drugs to reduce the expression of an operant ADE (Schroeder et al., 2005; Rodd et al., 2006; Dhaher et al., 2010). The results of our study showed that all DP doses tested (6.25, 12.5, or 25 mg/kg), intraperitoneally (i.p.) administered, were able to prevent the ADE in Wistar rats using an operant fixed ratio (FR) 1 procedure (Figure 1D). Contrarily to the saline group (named “DP 0”), DP blocked the increase in ethanol response following the imposed period of abstinence. Interestingly, animals treated with the higher DP dose (25 mg/kg) even reduced their response to ethanol significantly, by 20% below baseline levels. Moreover, DP did not modify the spontaneous motor activity of the rats indicating that the effectiveness of DP in preventing ADE cannot be due to a reduced locomotor performance of the animals (Martí-Prats et al., 2015). These results added reproducibility and robustness to previously reported data. Hence, to sum up, we were able to replicate our previous outcomes in a different laboratory (Laboratory of Psychobiology, Complutense University of Madrid) and using a different paradigm, (inter-lab reliability) leading to more robust conclusions on the use of DP as a potential new pharmacotherapy in the treatment of alcoholism.

### Effect of DP on Voluntary Ethanol Consumption Behavior

As illustrated above, findings of different research groups working in the field agree with the efficacy of DP as a valid strategy to prevent alcohol relapse-like drinking behavior, denoting the relevant role of ACD on the relapse expression. Conversely, concerning voluntary ethanol intake, the few published studies reveal contradictory results on the efficacy of DP (Font et al., 2006b; Campos-Jurado et al., 2015; Gosalbez et al., 2015).

Font et al. (2006b) were the first group to evaluate the effect of DP on voluntary ethanol consumption. In their study, male Long-Evans rats had daily access to a 10% ethanol solution in their home-cages for a 15-min period. Under their experimental conditions, the systemic (50 and 75 mg/kg), as well as ICV (75 μg) administration of DP was able to decrease the ethanol intake during the 5-day treatment. Interestingly, after discontinuation of the treatment, animals recovered their previous consumption rates. These results represent the initial evidence of ACD sequestration usefulness as a possible valid strategy to prevent ethanol drinking. However, in the same study, the authors also showed that systemic, but not ICV, DP treatment modified sucrose intake. According to the taste reactivity test performed, authors attributed this effect to a modification of ethanol palatability due to DP administration. Thus, all these results suggest that, although part of the DP effect on modulating ethanol consumption could be ascribed to a taste modification, an effect on the central levels of ACD has also been demonstrated (Font et al., 2006b).

Since the abovementioned study, it was not until nearly 10 years later that new research evaluating the utility of DP on the voluntary ethanol intake was performed. Concretely, Peana et al. (2015) studied the validity of DP on the acquisition and maintenance of oral operant ethanol self-administration. For acquisition analysis, ethanol-naïve Wistar rats were i.p. administered with DP (50 mg/kg) concomitant with the access to ethanol solution under an FR-1 schedule of reinforcement. The ethanol concentration was gradually increased from 5% in the first three sessions to 10% in the eighth last session. In this phase, systemic DP treatment significantly reduced the number of ethanol nose pokes, consistent with an ethanol intake decrease from the second ethanol session until the end of the study. Nevertheless, when rats, after an acquisition period, self-administered 10% ethanol for at least 10 days, the same DP treatment (50 m/kg) failed to reduce active nose pokes for ethanol. Indeed, the double dose of DP (100 mg/kg) was also unable to diminish ethanol self-administration. Furthermore, DP 50 mg/kg did not diminish the ethanol intake when the solution was changed from 10% to 5%, neither alone nor in combination with the catalase inhibitor amino-1,2,4-triazole (1 g/kg). Curiously, this catalase inhibitor, that has shown to be effective in reducing ethanol consumption during the acquisition phase, was ineffective along the maintenance phase (Peana et al., 2015). Hence, these results confirm the DP efficacy in impairing the acquisition of ethanol self-administration in naïve animals, but not in reducing active responses in ethanol-experienced animals. Indeed, ethanol-experienced rats increased their nose pokes when the ethanol concentration was reduced from 10% to 5% in order to obtain the same ethanol intake. According to these authors, a possible explanation for the failure of DP activity could be that ACD, paradoxically contributes to the perpetuation of ethanol self-administration, concretely the reduction of ACD levels due to the administered treatment might motivate rats to further seek and take ethanol to compensate for that decrease. Moreover, according to the abovementioned authors, additional explanations could be plausible since in these experimental conditions, due to its mechanism of action, DP would allow the increase of the non-metabolized fraction of ethanol in relation to the metabolized fraction (ACD and its derivatives). Under this condition, some studies have reported that ethanol, through the GABA_A_ receptor, might lead to different behavioral responses such as decreasing the rat’s locomotor activity (Martí-Prats et al., 2015) or increasing the responses in the paradigm of alcohol self-administration (Kumar et al., 2009; Kaminski et al., 2013).

**Figure 2:**
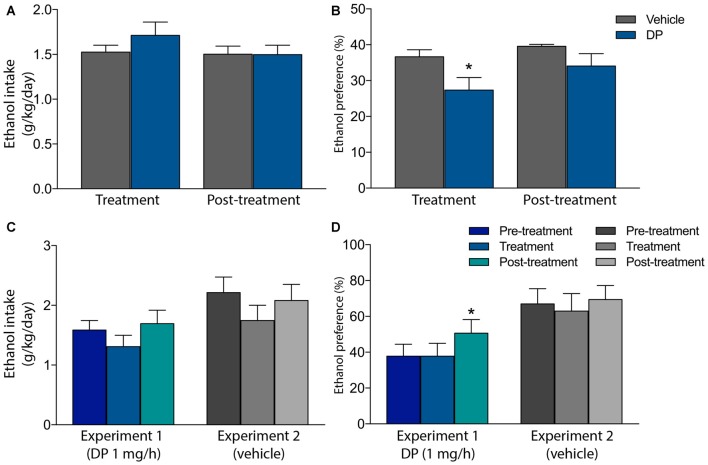
**Effect of systemic administration of DP on voluntary ethanol intake and alcohol preference in ethanol-naïve (A,B)** or in long-term ethanol-experienced rats **(C,D)**. **(A)** DP does not modify voluntary ethanol intake in ethanol-naïve rats **(B)** DP reduced ethanol preference in ethanol-naïve rats. In panels **(A,B)**, animals received DP (1 mg/h) or vehicle, from a mini-osmotic pump subcutaneously (SC) implanted, along the treatment phase. Along the post-treatment phase no substance was administered. Asterisk denotes statistically significant differences with regard to the vehicle group (*p* < 0.05). **(C)** DP does not modify voluntary ethanol intake in long-term ethanol experienced rats. **(D)** DP does not modify ethanol preference in long-term ethanol experienced rats. A change in ethanol preference was detected after DP treatment only. In panels **(C,D)**, animals received DP (1 mg/h) or vehicle, from a mini-osmotic pump SC implanted, only along the treatment phase. Along the pre-treatment and post-treatment phase no substance was administered. Asterisk denotes statistically significant differences relative to the pre-treatment and treatment phases (*p < 0.05; Adapted from Campos-Jurado et al., 2015; Gosalbez et al., 2015).

Finally, two additional pre-clinical studies have recently evaluated the ability of DP to prevent voluntary ethanol consumption, in both ethanol-naïve and ethanol-experienced Wistar rats, in their home-cages and under non-operant procedures (Campos-Jurado et al., 2015; Gosalbez et al., 2015). In the former, animals were SC implanted with a mini-osmotic pump delivering either vehicle or DP 1 mg/h along a 1-week period. One day after surgery, every animal was exposed in its home-cage, for the first time, to a four-bottle alcohol self-administration model. Along the next 6 days of treatment, although DP did not modify the voluntary ethanol intake, a significant reduction in the preference for ethanol, with respect to total volume of consumed liquid, was detected in the animals receiving DP. No statistical differences were detected in the next 6 days after treatment (post-treatment phase) between both experimental groups (Figures 2A,B).

With regard to the ethanol-experienced rats, animals were exposed to ethanol, under a non-operant paradigm during a 14-week period. Next, ethanol intake was registered on a daily basis along 3 weeks, each week corresponding to a different experimental period: (i) Pre-treatment: baseline intake was established; (ii) Treatment: a mini-osmotic pump was implanted in animals delivering DP 1 mg/h along 1 week; (iii) Post-treatment: the mini-osmotic pump was removed. Thereafter, rats continued to drink freely for 2 weeks before repeating the same experimental procedure, although pumps delivered sterilized water (vehicle) instead of DP. In this study, no differences in ethanol consumption nor ethanol preference were reported when animals received 1 mg/h DP treatment (Figures 2C,D). Nevertheless, after DP was administered (post-treatment phase), rats increased their ethanol preference in relation to pre-treatment and treatment phases, however, ethanol intake remained unmodified. Yet, these data suggest that, at the tested DP dose, a change in the consumption pattern takes place, without altering the total ethanol consumption.

Although different studies denote the existence of a positive correlation between brain ACD levels and alcohol intake (Correa et al., 2012; Muggironi et al., 2013; Israel et al., 2015), some aspects still remain unclear. In this sense, the inhibition of the brain ACD formation has been related to a reduction in the voluntary ethanol intake in mice and rats (Aragon and Amit, 1985, 1992; Koechling and Amit, 1994; Karahanian et al., 2011; Quintanilla et al., 2012; Ledesma et al., 2014). Moreover, the increase of the ACD metabolism (Karahanian et al., 2015) or the reduction of ACD disposition (Font et al., 2006b; Peana et al., 2010) has also been associated with a significant inhibition of voluntary ethanol consumption. Conversely, and consistent with some of the results exposed herein, different groups have reported that several strategies aimed at reducing the ACD levels in the brain, inhibition of its formation (Quintanilla et al., 2012; Tampier et al., 2013; Karahanian et al., 2015; Peana et al., 2015) or, as shown before, ACD inactivation (Campos-Jurado et al., 2015; Gosalbez et al., 2015), have not been able to impair the ethanol intake when experimental animals have moderate to long ethanol-experience. Recently, some studies have suggested a differential influence of ACD on the ethanol intake. It has been proposed that the key role of ACD in the ethanol reinforcing properties could be limited to the initial ethanol experience (called *first hit*), while after this first phase, ethanol consumption may not depend on ACD levels (Israel et al., 2015; Quintanilla et al., 2016). However, alternative explanations in relation with divergent results have also been taken into account. On the one hand, some of the studies supporting the preceding theory (Quintanilla et al., 2012; Karahanian et al., 2015) have focused on the manipulation of ACD levels only in the VTA, leaving open the possibility that other brain areas could be involved in the maintenance of alcohol intake (Karahanian et al., 2015). On the other hand, ethanol-related cues could also support the perpetuation of ethanol drinking behavior independent of its reinforcing properties (Greeley et al., 1993; Miller and Gold, 1994; O’Brien et al., 1998; Tiffany and Carter, 1998; Everitt et al., 2001; Littleton et al., 2001; See, 2002; Ingjaldsson et al., 2003a,b; van de Laar et al., 2004; Weiss, 2010; Karahanian et al., 2015; Peana et al., 2015).

Taking all the data into consideration, several of the hypotheses exposed could be due to the different data obtained. However, the diversity of protocols, experimental animals, rodent strains and duration of ethanol exposition make it difficult to realistically compare the results obtained in the published studies hitherto. Hence, further studies are required to obtain a firmer conclusion about DP efficacy in relation with voluntary ethanol intake.

## Conclusive Remarks and Future Directions

To sum up, all of these experimental findings provide the rationale for using ACD sequestering agents, concretely DP, as a novel therapeutic intervention alone and or in combination to prevent relapse into alcohol seeking and consumption. In fact, there is a consensus among researchers that this potential therapeutic avenue deserves more attention and investigation (Melis et al., 2007; Sanchis and Aragón, 2007; Enrico et al., 2009; Peana et al., 2010; Orrico et al., 2013, 2014). This is also based on the fact that this drug acts as an ACD-scavenging compound (Nagasawa et al., 1980) without altering any neurotransmitter systems (Salaspuro et al., 2006). Hence, we hypothesized that DP could be a promising drug for preventing alcohol relapse. On the other hand, its effectiveness in reducing voluntary ethanol consumption in long-term experienced patients still remains unclear. In this sense, in our opinion, one possibility that could be clinically explored is its administration “as needed” in agreement with the latest trends in therapy such as with Nalmefene. Additionally, another point in favor of this strategy is the fact that DP has already been approved for its use in humans, hence, this fact would lead to a faster and easier way of inclusion in the present limited therapeutic arsenal. In conclusion, there is a vast amount of pre-clinical research that demonstrates the potential use of DP for treating alcoholism. At this point, it is time for clinical researchers to try to cross the so-called “Valley of Death” for alcohol drug development, i.e., the gap between the demonstrated efficacy in pre-clinical animal models and clinical testing. This gap has impeded several promising novel compounds from moving forward along the drug development pipeline (Litten et al., 2012).

## Author Contributions

AO and LM-P wrote the review. MJC-C and LG made substantial contributions to the design of the work and interpretation of data for the work. AP and TZ conceptualized and drafted the work critically for important intellectual content. All authors revised and approved the final version.

## Funding

The research performed to elaborate this work was funded by grants from Conselleria de Educación (Generalitat Valenciana GVAICO2016-096), Universitat de València (UV-INV-AE11-42811) and (UV-INV-AE15-336743) (Spain) and Ministerio de Sanidad y Política Social (PND2009-021).

## Conflict of Interest Statement

The authors declare that the research was conducted in the absence of any commercial or financial relationships that could be construed as a potential conflict of interest. The reviewer LF and handling Editor declared their shared affiliation, and the handling Editor states that the process nevertheless met the standards of a fair and objective review.
